# Methanogens and Iron-Reducing Bacteria: the Overlooked Members of Mercury-Methylating Microbial Communities in Boreal Lakes

**DOI:** 10.1128/AEM.01774-18

**Published:** 2018-11-15

**Authors:** Andrea G. Bravo, Sari Peura, Moritz Buck, Omneya Ahmed, Alejandro Mateos-Rivera, Sonia Herrero Ortega, Jeffra K. Schaefer, Sylvain Bouchet, Julie Tolu, Erik Björn, Stefan Bertilsson

**Affiliations:** a Department of Ecology and Genetics, Limnology and Science for Life Laboratory, Uppsala University, Uppsala, Sweden; b Department of Marine Biology and Oceanography, Institut de Ciències del Mar, Consejo Superior de Investigaciones Científicas, Barcelona, Catalonia, Spain; c Department of Forest Mycology and Plant Pathology, Science for Life Laboratory, Swedish University of Agricultural Sciences, Uppsala, Sweden; d Department of Environmental Sciences, Rutgers University, New Brunswick, New Jersey, USA; e Department of Chemistry, Umeå University, Umeå, Sweden; f Department of Ecology and Environmental Science, Umeå University, Umeå, Sweden; University of Bayreuth

**Keywords:** mercury, methylation, *hgcA* gene, 16S rRNA gene, boreal lakes, methanogens, iron-reducing bacteria, sulfate-reducing bacteria

## Abstract

Methylmercury is a potent human neurotoxin which biomagnifies in aquatic food webs. Although anaerobic microorganisms containing the *hgcA* gene potentially mediate the formation of methylmercury in natural environments, the diversity of these mercury-methylating microbial communities remains largely unexplored. Previous studies have implicated sulfate-reducing bacteria as the main mercury methylators in aquatic ecosystems. In the present study, we characterized the diversity of mercury-methylating microbial communities of boreal lake sediments using high-throughput sequencing of 16S rRNA and *hgcA* genes. Our results show that in the lake sediments, Methanomicrobiales and Geobacteraceae also represent abundant members of the mercury-methylating communities. In fact, incubation experiments with a mercury isotopic tracer and molybdate revealed that only between 38% and 45% of mercury methylation was attributed to sulfate reduction. These results suggest that methanogens and iron-reducing bacteria may contribute to more than half of the mercury methylation in boreal lakes.

**IMPORTANCE** Despite the global awareness that mercury, and methylmercury in particular, is a neurotoxin to which millions of people continue to be exposed, there are sizable gaps in the understanding of the processes and organisms involved in methylmercury formation in aquatic ecosystems. In the present study, we shed light on the diversity of the microorganisms responsible for methylmercury formation in boreal lake sediments. All the microorganisms identified are associated with the processing of organic matter in aquatic systems. Moreover, our results show that the well-known mercury-methylating sulfate-reducing bacteria constituted only a minor portion of the potential mercury methylators. In contrast, methanogens and iron-reducing bacteria were important contributors to methylmercury formation, highlighting their role in mercury cycling in the environment.

## Abstract (precis)

Despite the global awareness that mercury, and methylmercury in particular, is a neurotoxin to which millions of people continue to be exposed, there are sizable gaps in the understanding of the processes and organisms involved in methylmercury formation in aquatic ecosystems. In the present study, we shed light on the diversity of the microorganisms responsible for methylmercury formation in boreal lake sediments. All the microorganisms identified are associated with the processing of organic matter in aquatic systems. Moreover, our results show that the well-known mercury-methylating sulfate-reducing bacteria constituted only a minor portion of the potential mercury methylators. In contrast, methanogens and iron-reducing bacteria were important contributors to methylmercury formation, highlighting their role in mercury cycling in the environment.

## INTRODUCTION

Mercury (Hg), a ubiquitous and naturally occurring element in the environment, is considered a priority hazardous substance because of its high toxicity (1). Humans have emitted Hg to the atmosphere for millennia (2). Long-range atmospheric transport of released Hg is accumulating in ecosystems globally, and this has led to increased Hg levels in surface (atmosphere, oceans, and terrestrial) reservoirs (3, 4). This is of special concern in the boreal biome, since a large pool of Hg has accumulated in soils following atmospheric deposition during the industrial era (5). As Hg binds strongly to organic matter (OM), the release of OM from soils to aquatic systems also affects the fate of Hg in boreal catchments (6). For example, increased import of terrigenous OM from surrounding catchments, due to ongoing climate change (7), may lead to higher Hg levels in boreal lakes (8, 9). As a result, millions of people are exposed to harmful levels of this potent neurotoxin via fish consumption (10, 11). This health hazard especially concerns organic methylmercury (MeHg) which bioaccumulates in organisms and is biomagnified in aquatic food webs (12, 13). A deeper understanding of the processes, organisms, and environmental conditions involved in MeHg production in aquatic ecosystems may thus help us develop more efficient management strategies for limiting human MeHg exposure.

In aquatic ecosystems, the methylation of divalent inorganic Hg [Hg(II)] to MeHg has mainly been attributed to the action of sulfate-reducing bacteria (SRB) (14–17) and, in some cases, to iron-reducing bacteria (FeRB) (18, 19), methanogens (20), and syntrophs (21). Previous studies have relied on inferences from soil phospholipid fatty acid analysis (22, 23), taxonomic markers (e.g., 16S rRNA genes [20, 24]), or specific functional genes involved in sulfate reduction (e.g., *dsrAB*) to indirectly describe the composition of putatively Hg(II)-methylating microbial communities (16, 25–27). However, the recent discovery of two functional genes, *hgcA* and *hgcB*, which play essential roles in Hg(II) methylation (28), opens the possibility to use direct markers for Hg(II)-methylating organisms in complex communities. Indeed, the recent use of *hgcA* and *hgcB* genes has greatly improved the ability to describe Hg(II)-methylating microbial communities at a higher taxonomic resolution (21, 29–31). Despite recent studies of Hg methylators in wetlands (21, 29, 30), the diversity of Hg(II)-methylating microbial communities and the factors shaping these communities in freshwater environments remain largely unexplored.

Temperature, redox, and pH are known to be important geochemical factors regulating Hg(II) methylation (32). Furthermore, a recent study showed that the molecular composition of organic matter (OM) controlled Hg(II) methylation in boreal lake sediments (9). This study revealed that sediments enriched in autochthonous plankton-derived OM presented high Hg(II) methylation rates, associated with enhanced bacterial activity. Specific OM compounds can also promote Hg(II) methylation by regulating Hg(II) speciation (33) and Hg(II) availability (34–36). OM can also facilitate Hg(II) methylation by enhancing mercury sulfide (HgS) dissolution or inhibiting HgS precipitation, thereby providing available Hg(II) for methylating microorganisms (37). In contrast, high OM concentrations might also decrease Hg methylation through the formation of high-molecular-mass complexes that limit Hg(II) availability (34). Altogether, these studies highlight that both geochemical conditions and OM composition are central regulators of microbial Hg(II) methylation. In the present study, we provide a detailed characterization of Hg(II)-methylating microbial communities of boreal lake sediments featuring contrasting OM molecular compositions (9). This was accomplished using a combination of experimental incubations and field surveys based on high-throughput sequencing of 16S rRNA and *hgcA* genes. In doing so, we implicate methanogens and iron-reducing bacteria as overlooked mediators of this process in boreal lake sediments, and we identified an important link between the composition of Hg(II)-methylating communities and the degradation status of phytoplankton-derived OM in 10 lakes with contrasting trophic statuses (Table 1).

**TABLE 1:** Characteristics of the investigated lakes

Lake	Sample code	Coordinates (°)		Geochemical parameters of the sediment overlying water^*a*^									
		N	E	*z* (m)	pH	Temp (°C)	C (μS · s^−1^)	O_2_ concn (mg · liter^−1^)	DOC concn (mg · liter^−1^)	TP concn (μg · liter^−1^)	Chla concn (μg · liter^−1^)	SO_4_^2−^ concn (mg · liter^−1^)	SUVA_254_ (liters · mg^−1^ · m^−1^)
Lilla Sångaren	LS	59.8996	15.3923	17.0	6.9	5	60	4.65	7.0	23	1.8	2.9	4.21
Ljustjärn	LJU	59.92375	15.453472	10	7.3	6.9	77	0.22	6.5	96	93	2.2	1.6
Svarttjärn	S	59.89073	15.2577	6.5	5.6	4.8	59	0.08	22.0	36	2.7	0.9	5.96
Fälaren	F	60.33656	17.79396	2.0	7.5	18.7	67	8.56	32.6	20	8.9	3.0	3.9
Oppsveten	O	59.98874	15.57562	10.0	6.3	8.6	30	0.79	16.7	14	Udl^*b*^	2.3	4.78
Strandsjön	STR	59.87099	17.168650	2.5	6.9	16.4	285	0.34	19.6	60	13.1	4.6	3.0
Valloxen	V	59.73846	17.83954	6.0	8.5	18.8	502	0.14	12.3	49	52	8.9	2.5
Vallentunasjön	VALE	59.50435	18.037083	4	7.1	17.2	469	0.24	14.0	77	58	16.2	1.4
Marnästjärn	M	60.14483	15.20714	2.0	7.2	17.8	185	6.30	9.2	185	190	3.6	1.8
Lötsjön	LOTS	59.86314	17.940110	7	6.8	11.5	288	0.3	13.3	65	18	1.60	1.7

a C, conductivity; O_2_, oxygen concentration; DOC, dissolved organic carbon; TP, total phosphorus; Chla, chlorophyll *a*; SO_4_^2−^, sulfate; SUVA_254_, specific UV absorbance of the water overlying the sediment (modified from reference 9).

b Udl, under detection limit.

## RESULTS

### Hg(II)-methylating microbial communities.

**FIG 1:**
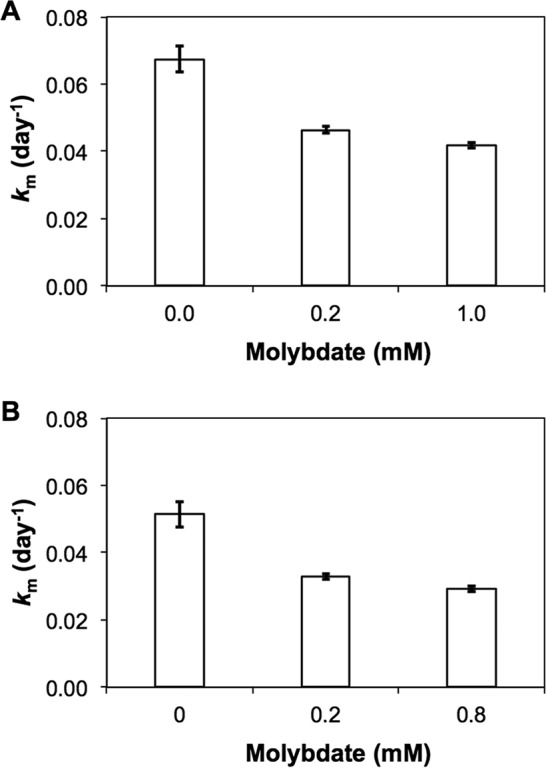
Hg(II) methylation (day^−1^) obtained in unamended (0 mM) and molybdate-amended sediments (3 replicate incubation experiments) for Valloxen (A) and Marnästjärn (B) lakes.

The relative contribution of sulfate reducers to mercury methylation was assessed by adding molybdate and an enriched stable isotope (^198^HgCl_2_) to sediments of lakes known to have high Hg(II) methylation rate constants (*k*_m_; lake M and lake V) (Table 2). The addition of molybdate inhibited the *k*_m_ by 38% and 45%, respectively (Fig. 1). This partial inhibition suggests that SRB were not the only significant Hg(II) methylators in the studied sediments.

**TABLE 2:** Concentrations of Hg(II) and MeHg, Hg(II) methylation yields, total carbon, carbon to nitrogen ratio, total phosphorous, and bacterial production of the investigated lake sediments

Lake	Sediment characteristics^*a*^						
	Hg(II) (ng · g^−1^)	MeHg (ng · g^−1^)	*k*_m_ (day^−1^)	TC (%)	C/N	TP (%)	BP (μg C · liter^−1^ · day^−1^)
LS	231 ± 31	6.6 ± 0.9	0.0095 ± 0.0049	19.1 ± 1.0	15.1 ± 0.018	0.018 ± 0.0020	4.0 ± 0.44
LJU	261 ± 8	4.0 ± 0.3	0.0095 ± 0.0007	31.9 ± 0.2	12.9 ± 0.012	0.012 ± 0.0009	0.4 ± 0.66
S	373 ± 11	7.2 ± 1.1	0.0110 ± 0.0001	23.7 ± 0.8	16.0 ± 0.011	0.011 ± 0.0003	2.7 ± 0.04
F	235 ± 5	3.9 ± 0.2	0.0120 ± 0.0071	24.4 ± 0.3	12.5 ± 0.010	0.010 ± 0.0009	6.4 ± 1.87
O	253 ± 49	6.4 ± 0.4	0.0125 ± 0.0021	19.2 ± 0.3	17.5 ± 0.013	0.013 ± 0.0010	1.9 ± 0.37
STR	179 ± 1	1.4 ± 0.1	0.0130 ± 0.0028	11.7 ± 0.1	8.7 ± 0.014	0.014 ± 0.0008	16.6 ± 0.25
V	74 ± 1	2.5 ± 0.5	0.0775 ± 0.0007	13.9 ± 0.2	8.1 ± 0.015	0.015 ± 0.0000	23.6 ± 3.43
VALE	102 ± 8	2.0 ± 0.1	0.0590 ± 0.0099	18.9 ± 0.2	7.9 ± 0.018	0.018 ± 0.0001	21.3 ± 2.29
M	12,711 ± 158	100 ± 5.7	0.0385 ± 0.012	19.7 ± 0.3	9.1 ± 0.024	0.024 ± 0.0005	14.6 ± 1.93
LOTS	156 ± 8	2.6 ± 0.3	0.0780 ± 0.0014	14.3 ± 0.6	8.2 ± 0.021	0.021 ± 0.0004	17.2 ± 2.28

a TC, total carbon; C/N, carbon-to-nitrogen ratio; TP, total phosphorus; BP, bacterial production. Data are means from two depths (0 to 1 cm and 1 to 2 cm). Modified from reference 9.

**FIG 2:**
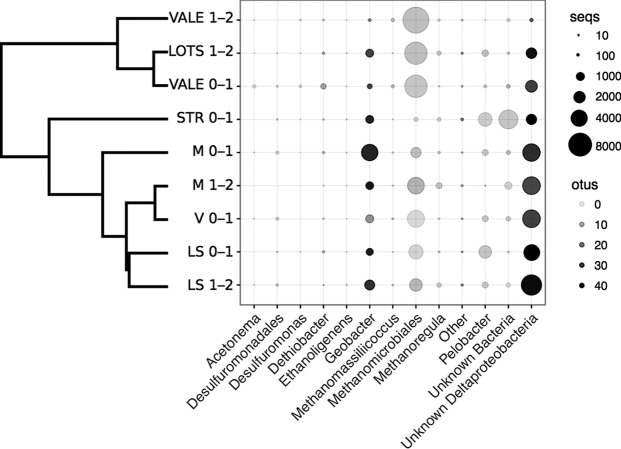
Hg(II)-methylating microbial community compositions of boreal lake sediments (two depths: 0 to 1 cm and 1 to 2 cm) based on the Hg(II) methylation gene (*hgcA*) sequencing at a highly resolved taxonomic level. The sizes of the symbols illustrate the relative abundance of each taxon. Shading of the symbols (gray scale) represents the number of individual operational taxonomic units (OTUs; 80% similarity threshold) within the taxa. By using hierarchical clustering, the dendrogram to the left demonstrates the community similarity between samples.

As our incubation experiments revealed Hg(II)-methylating activity beyond that of sulfate reducers, we focused on the identification of other organisms mediating this process. For this, we analyzed the compositions of the Hg(II)-methylating microbial communities by high-throughput sequencing of amplified *hgcA* genes. We detected the *hcgA* gene in nine samples from six of the ten lakes (Fig. 2), including samples from all four lakes with high Hg(II) methylation rate constants (VALE, V, LOTS, and M, *k*_m_ > 0.02 day^−1^) and two lakes with low *k*_m_s (STR and LS, *k*_m_ < 0.02 day^−1^). The total microbial *hgcA* gene data set consisted of 78,642 reads distributed across 255 operational taxonomic units (OTUs). Of these, 224 were related to Bacteria, 25 to Archaea, and 6 remained unknown. Most of the Hg(II) methylator diversity targeted by the primers used was captured in our analyses (see Fig. S1 in the supplemental material). Deltaproteobacteria accounted for 48% of the reads, followed by Methanomicrobia with 42.1% of the reads and Clostridia with 0.82% of total reads (Fig. 2). Reads that could not be assigned to any known taxonomic group (unknown bacteria) comprised 7.2% of the total reads. Within Deltaproteobacteria, 30.5% of the reads were assigned as unknown Deltaproteobacteria, while 18% of the reads were affiliated with Desulfuromonadales. Approximately half of the Desulfuromonadales reads were affiliated with the FeRB Geobacteraceae (9.8% of total reads) and the other half affiliated with Pelobacteraceae (Desulfuromonadaceae; 7.2% of total reads) species. Well-known sulfate-reducing Hg(II) methylators such as Desulfovibrionales represented only 0.08% of the total reads despite the high efficiency of these PCR primers to amplify *hgcA* from cultured representatives within the Desulfovibrionales relative to that from other orders (29, 38).

**FIG 3:**
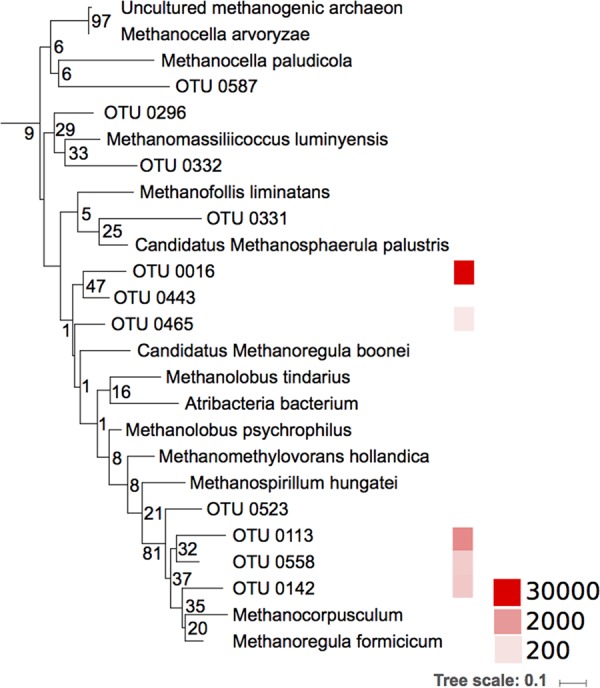
Phylogenetic distribution of the Hg(II) methylation gene (*hgcA*) sequences from archaeal methanogens, including both reference strains and environmental sequences from boreal lake sediments. The tree was generated using RAxML (version 8.2.4) with the PROTGAMMLG model and the autoMR to choose the number of necessary bootstraps (750). The colors to the right illustrate the abundance of the sequences (a total of 78,462 sequences). Scale bar represents estimated phylogenetic distance in substitutions per site, and the numbers at the branch points indicate the bootstrap values.

**FIG 4:**
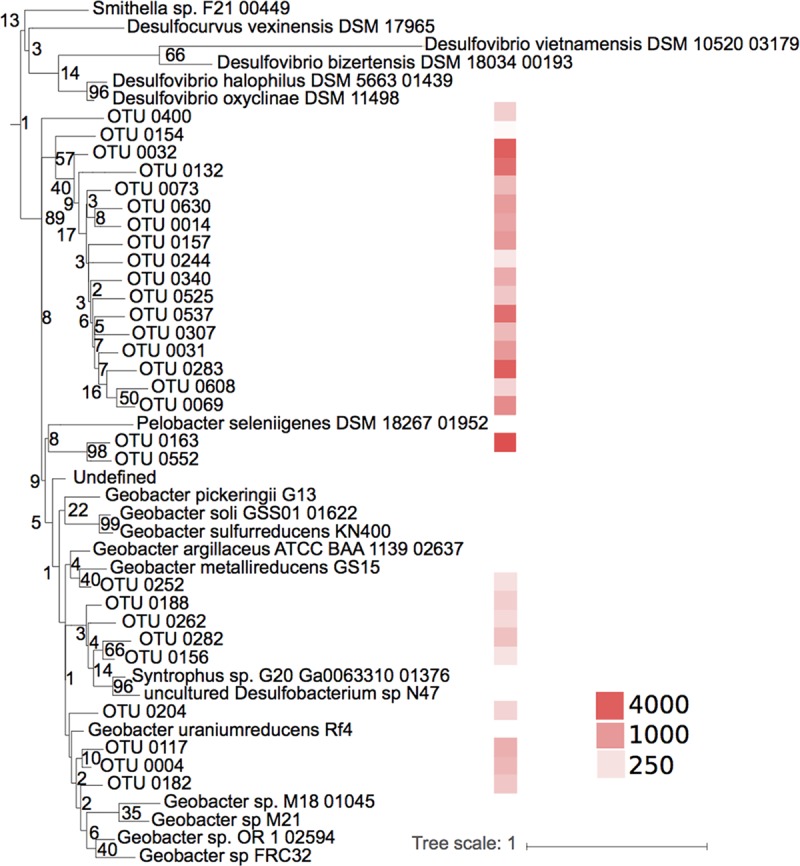
Maximum likelihood tree and abundance of the Hg(II) methylation gene operational taxonomic units (OTUs) closely related to a Pelobacter sp. and Geobacter spp. from boreal lake sediments. The colors on the right illustrate the abundance of the sequences (a total of 78,462 sequences). Scale bar represents estimated phylogenetic distance in substitutions per site, and the numbers at the branch points indicate the bootstrap values.

The phylogenetic analyses demonstrated that some of the unidentified OTUs in this study were closely related to Hg(II)-methylating methanogens (Fig. 3) and to Geobacteraceae and Desulfuromonadaceae families (Fig. 4). A substantial Hg(II) methylation activity could also be mediated by hitherto unknown Hg(II)-methylating organisms that could not be taxonomically resolved beyond the class level. This is the case for many of the OTUs classified merely as Deltaproteobacteria. To illustrate the lack of reference information, we placed the representative sequences of the most abundant unclassified *hgcA* gene OTUs in a phylogenetic tree to show their relationships to already described genes from genomes and isolates (see Fig. S2). These OTUs were abundant and also featured a high diversity. Several of the unknown Deltaproteobacteria OTUs found in our study were phylogenetically related to the SRB Desulfovibrio oxyclinae, indicating that unknown Deltaproteobacteria OTUs with Hg(II)-methylating capacity are also likely to be abundant in boreal lake sediments.

### Bacterial community composition.

**FIG 5:**
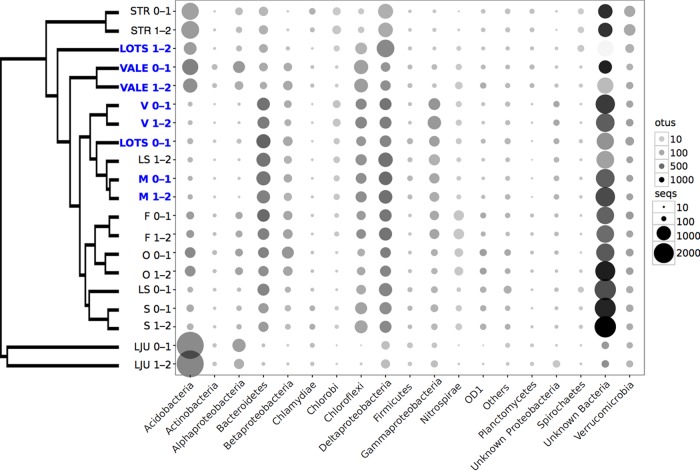
Compositions of sediment bacterial community across boreal lake sediments based on 16S rRNA gene amplicon analysis. Symbol sizes illustrate the abundance of the organisms at the phylum level, except for Proteobacteria (class level). Symbol shade (gray scale) represents the number of individual operational taxonomic units (OTUs; 97% similarity threshold) within each lineage. The dendrogram to the left shows the results of a cluster analysis and highlights community similarities between individual samples. Lakes with high Hg(II) methylation rate constants (*k*_m_ > 0.02 day^−1^) are highlighted in blue boldface font.

As Hg(II)-methylating communities coexist with other microorganisms in sediments, we also described the compositions of the sediment bacterial communities to assess potential interactions and coupling between Hg(II)-methylating communities and the overall bacterial communities. The bacterial 16S rRNA gene data set contained 258,020 high-quality reads, which grouped into 38,681 OTUs at 97% sequence identity. The data were normalized to the lowest number of reads for any individual sample, resulting in 6,045 reads per sample and a total of 23,485 OTUs. In general, the bacterial communities of the samples collected in the same lake at different depths (0 to 1 cm and 1 to 2 cm) were highly similar (see Fig. S3). The most abundant phyla were Acidobacteria (15.0%), Proteobacteria (12.7%, almost exclusively represented by Deltaproteobacteria), Bacteroidetes (9.4%), and Chloroflexi (8.0%) (Fig. 5). These phyla are often seen in freshwater sediment communities (39–42). Except for lake LJU, the distributions of major taxa were similar across the full set of lakes, with phylum Acidobacteria and unknown bacteria exhibiting the largest variation in abundance (see Fig. S4). Bacterial alpha-diversity, measured as the number of observed OTUs, varied between samples (from 804 to 2,723 OTUs), and also between phyla (Fig. 5), with Acidobacteria (1,242 OTUs; 13.6% of total reads) representing a particularly abundant phylum (Fig. 5), followed by Bacteroidetes (1,610 OTUs; 9.2% of total reads), Chloroflexi (988 OTUs; 12.4% of total reads), and Deltaproteobacteria (1,944 OTUs; 7.9% of total reads). Within the Deltaproteobacteria, OTUs related to known Hg(II) methylators, i.e., orders Syntrophobacterales, Desulfobacterales, Desulfuromonadales, and Desulfovibrionales accounted for 4.7%, 1.0%, 0.8% and 0.01% of the reads, respectively, across the full set of samples. Families Syntrophaceae and Syntrophobacteraceae accounted for 4.2% and 0.4%, respectively, of Syntrophobacterales. Within the Firmicutes, the order Clostridiales contributed 0.7% of the total reads, half of them belonging to the family Ruminococcaceae.

With regard to the overall composition of microbial communities, there seems to be coherence between *hgcA* and 16S rRNA genes (correlation in a symmetric Procrustes rotation, 0.67; *P* = 0.034). However, the community compositions between samples collected from the same lake were more uniform for 16S rRNA gene data than for the *hgcA* gene data (see Fig. S5). However, there was no direct link between the total bacterial community composition and Hg(II) methylation rate constants in the studied lake sediments (permutational multivariate analyses of variance [PerMANOVAs], *P* = 0.139, *F* = 1.46, *R*^2^ = 0.075). Indeed, lakes with highly similar bacterial community compositions, as for example, LS0-1 and M0-1 (Fig. 5), presented very different Hg(II) methylation rate constants (Table 2).

### Effects of OM composition and resident bacterial communities on the composition of Hg(II)-methylating microorganisms.

**FIG 6:**
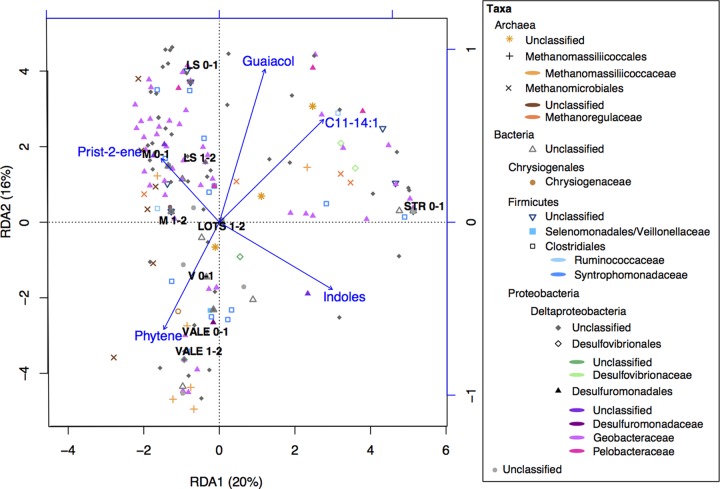
RDA triplot (2 first components) of the Hg(II)-methylating microbial community (*hgcA* gene, OTU level) (response variable) constrained by specific OM compounds (predicting variable) and the studied sites. The color coding in the legend indicates the highly resolved taxonomic levels of the Hg(II)-methylating community. The shapes correspond to the order level. The arrows and text summarize explanatory variables, corresponding to the descriptors of OM as follows: phytene, fresh phytoplankton-derived OM; prist-2-ene, degraded phytoplankton-derived OM; guaiacol, terrestrial OM; C_11–14:1_, degraded lipids; indoles, processed proteins.

In a previous study, Bravo et al. demonstrated that most of the variation in the OM composition was related to OM sources (terrestrial versus in-lake) and their degradation status (fresh versus degraded) (9). Here, we assessed the effect of OM molecular composition on the composition of Hg(II)-methylating microorganisms in boreal lake sediments by using the abundances of organic compounds previously identified by pyrolysis-gas chromatography-mass spectrometry (Py-GC/MS) (9). We used redundancy analysis (RDA), a well-recognized multivariate analysis method in microbial ecology (43), to regress a matrix of multiple response variables (i.e., the abundance of the OTUs for Hg(II)-methylating microorganisms) to a corresponding matrix of explanatory variables (i.e., the abundances of different OM compounds). However, RDA modeling has two prerequisites: (i) the number of pyrolytic organic compounds that can be included in the model must be lower than the number of observations (the 9 sediment samples), and (ii) the selected pyrolytic organic compounds should not be intercorrelated in order to optimize the precision in the prediction of the OTUs for Hg(II)-methylating microorganisms (43). In the final RDA model, the explanatory variables were composed of guaiacol, *n*-alkenes C_11_ to C_14_ (noted C_11–14:1_), prist-2-ene, phytene, and indoles (Fig. 6) (details on how these groups of pyrolytic organic compounds have been selected are provided in Materials and Methods). The RDA model (*F* = 1.5, *P* = 0.018) (Fig. 6) showed that those pyrolytic organic compounds explained 62% of the variation in the abundance of Hg(II)-methylating communities (RDA1 = 20%, RDA2 = 16%, RDA3 = 15%, RDA4 = 11%), with an associated *R*^2^ of 70%. More precisely, the Hg(II)-methylating community structure was significantly correlated with (listed in order of decreasing importance) indoles (*P* = 0.025), short *n*-alkenes (C_11–14:1_, *P* = 0.026), and prist-2-ene (*P* = 0.043). Both C_11–14:1_ and prist-2-ene are proxies of degraded OM. While the presence of indoles indicates protein processing, C_11–14:1_ and prist-2-ene denoted the presence of extensively degraded OM from lipids of multiple sources and chlorophylls or tocopherols (and thus from phytoplankton), respectively (44–46). In contrast, phytene and guaiacol are specific proxies for organic matter sources, namely, phytoplankton and terrestrially derived OM, respectively (44, 45, 47). Hence, the RDA suggests that the composition of Hg(II)-methylating communities is influenced by the degradation status of phytoplankton-derived OM. In contrast, the presence of terrigenous OM, for which guaiacol is a specific proxy, had smaller effects on the composition of Hg(II)-methylating communities in general (*P* > 0.05) (Fig. 6) and on the Hg(II) methylation rate constants of the studied lakes (Table 2) (9), as also suggested in the literature (48).

**FIG 7:**
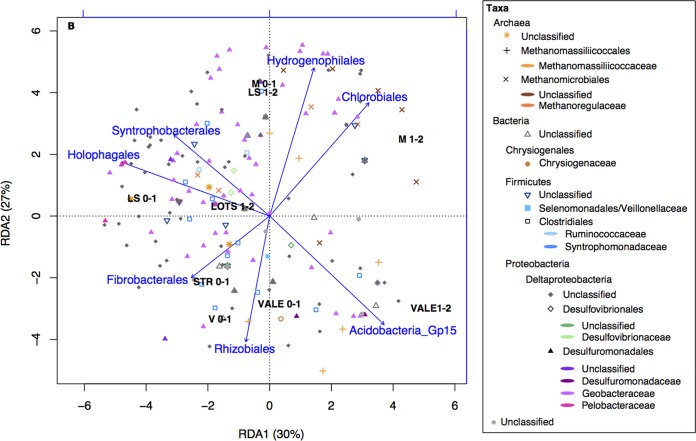
RDA triplot (2 first components) of the Hg(II)-methylating microbial community (*hgcA* gene, OTU level) (response variable) constrained by the resident bacterial community (16S rRNA gene) and the studied sites. The color coding in the legend indicates the highly resolved taxonomic levels of the Hg(II)-methylating community. The shapes correspond to the order level of the Hg(II)-methylating community. The arrows and text summarize explanatory variables, corresponding to taxa of resident bacterial community.

As sediments are habitats for a large number of organisms besides Hg(II) methylators, we also performed RDA to study the effect of the supporting and interacting bacterial communities on the composition of Hg(II) methylators (Fig. 7). The RDA identified a direct link between some specific groups of the sediment bacterial community and the composition of Hg(II)-methylating communities (*F* = 4.2, *P* = 0.005) (Fig. 7). The model explained 83.6% of the total variance (RDA1 = 30.1%, RDA2 = 26.8%, RDA3 = 16.1%, RDA4 = 10.6%) with an *R*^2^ of 96.7%. The percentage of accumulated constrained eigenvalues of the axes explained 31.1%, 27.7%, 16.6%, and 10.9% of the variance, respectively. The RDA showed that the Hg(II)-methylating community structure was significantly correlated to the presence of (listed in order of decreasing importance) Syntrophobacterales (*P* = 0.001), Acidobacteria_Gp15 (*P* = 0.003), Chlorobiales (*P* = 0.005), Fibrobacterales (*P* = 0.006), Holophagales (*P* = 0.014), Hydrogenophilales (*P* = 0.02), and Rhizobiales (*P* = 0.030). This suggests dependences or a mutualistic relationship between the methylators and these abundant community members.

## DISCUSSION

### Relative contribution of different Hg(II)-methylating microbial guilds.

In this study, we found that SRB, considered primary Hg methylators in aquatic systems (14–16, 49), accounted for less than half of the Hg(II) methylation. Both the experiments with specific inhibitors and *hgcA* gene surveys demonstrated that the major part of the Hg(II) methylation (between 55% and 62%) *k*_m_ was most likely attributed to other functional groups, such as methanogenic Archaea and FeRB. The significance of methanogenic Archaea and FeRB as mediators for Hg(II) methylation has until recently been largely overlooked, but our incubations suggest that these metabolic guilds are important drivers of Hg(II) methylation. This is further supported by the abundance of *hgcA* reads affiliated with methanogens and FeRB (Fig. 1) in the data set. Methanogens were only recently revealed to play a role as Hg(II) methylators in experiments using pure cultures (50, 51), lake periphyton (20), and rice paddies (52) and by global *hgcA* gene inventories (29, 31, 53). The abundance of methanogens in the *hgcA* gene library (Fig. 1) and the phylogenetic analyses suggest that some of the unidentified OTUs in this study may represent hitherto unknown Hg(II)-methylating methanogens (Fig. 2).

It is also important to consider a potential indirect effect of molybdate amendments. Molybdate amendments might have decreased Hg(II) methylation rate constants not only by inhibiting the activity of Hg(II)-methylating SRB but also indirectly by interfering with syntrophic partnerships between Hg(II)-methylating methanogens and non-Hg(II)-methylating syntrophs (14), such as members of the family Syntrophaceae, that were abundant in the studied lakes.

Only a decade ago, FeRB were revealed as important mediators of Hg(II) methylation in sediments of Clear Lake (18) and, more recently, in sediments contaminated by sewage treatment plant effluents (19, 54), in paddy soils (30), and in wetlands (29). In addition to the reads specifically classified as belonging to Geobacteraceae, the phylogenetic analysis suggests that 11 OTUs (13% of the total reads) of the unknown Deltaproteobacteria may be close relatives of Desulfuromonadaceae and Geobacteraceae (Fig. 3). As many Geobacter spp. can methylate Hg(II) (53, 55), the abundance of Geobacteraceae, and relatives, in our *hgcA* data set suggests that FeRB are also putatively important members of Hg(II)-methylating communities of boreal lake sediments. The lack of specific inhibitors for Fe reduction limits our ability to quantify the specific contribution of FeRB to Hg(II) methylation rate constants relative to that from other groups and to determine whether Desulfuromonadaceae and Geobacteraceae methylate Hg(II) in Fe-reducing processes or might use alternative metabolic processes such as S^0^ reduction or syntrophic oxidation of OM (56).

Despite the expanded view that we provide on the diversity of Hg(II) methylators in boreal lake sediments, many of the putative Hg(II)-methylating microbial groups observed in the environment could not be robustly annotated according to metabolic type. Indeed, the SRB shown to mediate approximately 45% of MeHg formation were not robustly identified in the *hgcA* gene data set but were likely included in the large pool of OTUs classified merely as Deltaproteobacteria (see Fig. S2 in the supplemental material). Expanded genome-wide sequence databases for uncultured microbial lineages and further development and refinement of primers targeting *hgcA* and *hgcB* to specifically target different groups of Hg(II) methylators are thus needed to increase the comprehensive understanding of the distribution of Hg-methylating organisms in nature (38), including those in boreal lake sediments.

### Factors affecting the composition of Hg(II) methylators: the role of organic matter.

In the studied lakes, nonmetric multidimensional scaling (NMDS) plots of 16S rRNA and *hgcA* genes indicated that there was more spatial variation in the diversity of the *hgcA* gene than of the general bacterial 16S rRNA gene (Fig. S5). This implies that Hg(II)-methylating microbial communities might be more endemic or at least less homogenous than the overall bacterial community, possibly responding more strongly to the different environmental conditions experienced in the different lakes. Furthermore, the amount of bioavailable Hg(II), temperature, pH, and redox (57) as well as the composition of OM strongly influenced Hg(II) methylation processes (9). RDA model 1 revealed that the degradation status of phytoplankton-derived OM was an important factor controlling the composition of Hg(II)-methylating microorganisms (Fig. 6). In lakes with the highest Hg(II) methylation rate constants (V and VALE) (Table 2), fresh phytoplankton-derived OM (i.e., phytene) affected the composition of the Hg(II)-methylating communities toward a higher contribution of Geobacteraceae, Methanomassiliicoccales and unknown Deltaproteobacteria (Fig. 6). In contrast, degraded phytoplankton-derived OM (e.g., prist-2-ene) exerted strong control on Hg(II)-methylating microbial communities in the lakes featuring intermediate to low Hg(II) methylation rate constants (M and LS). In this last case, Hg(II)-methylating microbial communities were more diverse (Fig. 6). As Hg(II)-methylating activities are strain specific (23, 58), it is not surprising to find different Hg(II)-methylating strains from the same order in the different lakes.

An increase in nutrients (and subsequently primary production and phytoplankton-derived OM) has previously been linked to an enhancement of the anaerobic microflora in sediments (59). However, anaerobic Hg(II)-methylating communities only represent a subset of a larger microbial community involved in the transformation and recycling of organic and inorganic compounds in these habitats. Our results hint at an important role of the resident bacterial community in shaping the composition of the Hg(II)-methylating community in boreal lake sediments (Fig. 7). The anaerobic oxidation of OM from complex organic compounds generally goes through different steps and processes (60). After an initial hydrolysis of large organic substances (by, for example, Rhizobiales and Fibrobacterales), the degradation intermediates are fermented into smaller organic molecules, such as lactate, propionate, butyrate, acetate, and formate, as well as CO_2_ and H_2_ (by, for example, Holophagaceae). These fermentation products might then be used as electron donors for Desulfovibrionales, Geobacteraceae, and Syntrophobacterales that are known to contain Hg(II) methylators. One of the dominant products of OM oxidation is H_2_, which can be used by, for example, Hydrogenophilales. Many groups characterized by their capability to degrade OM, and therefore featuring a potential to impact Hg(II) methylation, were indicated in the RDA model 2 (Fig. 7). Thus, our results suggest a potential role of the non-Hg(II)-methylating bacteria involved in the OM decomposition cascade to fuel Hg(II)-methylating microbial communities.

The understanding of mechanisms explaining why some strains occur in one location but are absent in others is still a fundamental challenge in ecology (61). It is becoming increasingly clear that in general, environmental conditions are important for structuring the biogeography of bacterial communities (62). Hence, the differences in the distributions of Hg(II)-methylating taxa among the studied lakes might derive primarily from different species of the same family having different niche requirements (61). Our results suggest that a high relative abundance of phytoplankton-derived OM and the presence of specific strains of non-Hg(II)-methylating bacteria involved in OM decomposition create a niche that promotes Hg(II) methylation. We are in need of a more complete understanding of the biological pathways involved in Hg(II) methylation and the environmental factors controlling the presence and activity of the organisms mediating this process. In this context, our study provides some insights about the hitherto overlooked role of methanogens and FeRB in Hg(II) methylation in boreal lakes.

## MATERIALS AND METHODS

### Study area.

In this study, we studied 10 lakes with contrasting trophic statuses and receiving different amounts of terrestrial OM (Table 1). According to a previous study (9) and to the OM molecular compositions, these 10 lakes were grouped into two types: Lötsjön (LOTS), Marnästjärn (M), Strandsjön (STR), Vallentunasjön (VALE), and Valloxen (V) were dominated by autochthonous OM, whereas sediments from Svarttjärn (S), Ljustjärn (LJU), Lilla Sångaren (LS), Oppsveten (O), and Fälaren (F) were characterized by a predominance of allochthonous terrestrially derived OM. The lakes ranged from clear water to humic, with average dissolved organic carbon (DOC) concentrations ranging from 6.5 to 32.6 mg · liter^−1^, and from oligotrophic to hypereutrophic systems with chlorophyll *a* from below detection (<0.5 μg · liter^−1^) to 190 μg · liter^−1^ (Table 1). Conductivity and pH varied between 30 μS · s^−1^ and 469 μS · s^−1^ and between 5.6 and 8.7, respectively. Hg(II) methylation rate constants, determined by incubations with enriched isotopic tracers, varied from 0.9% to 7.8% (Table 2) (9). The details about sampling and sample handling have been described previously (9). Briefly, vertical summer profiles of water samples for chemical characterization were collected using a GoFlo bottle (polyvinyl chloride [PVC]). Some of the lakes were thermally stratified and presented oxygen-depleted waters overlying the sediment (Table 1). The sediment characteristics are presented in Table 2.

### Analytical methods.

Water samples were filtered through glass fiber filters (GF/F; Whatman, UK) and analyzed for sulfate, DOC, total phosphorus (TP), chlorophyll, and optical properties of the organic matter (Table 1). The DOC content from the water column was measured by high temperature catalytic oxidation (Shimadzu-TOC-L) (63). TP was analyzed according to Murphy and Riley (64). UV-visible absorbance spectra (200 to 800 nm) were measured with a Lambda 40 spectrophotometer (Perkin-Elmer) as previously described (65).

Sediment cores were collected using a 60-mm diameter gravity corer (Uwitec, Austria). Cores with approximately 40 cm of overlying water were kept upright at 4°C and processed within 12 h in a glove bag (Sigma-Aldrich, USA) under a N_2_ atmosphere. The water overlying the sediment was first retrieved with sterile syringes, and then the upper 2 cm of the sediment core (0 to 1 cm and 1 to 2 cm) were sliced using autoclaved sectioning tools. For each slice, a sediment subsample was immediately put in a sterile Cryotube and placed in liquid nitrogen for subsequent DNA extraction. Another subsample was immediately incubated for 1 h at *in situ* temperature after being spiked with ^3^H-labeled thymidine (Amersham, 1 mCi · ml^−1^, 80 Ci · mmol^−1^) to assess bacterial production (66). The last subsample was used to incubate sediments and determine Hg(II) methylation rate constants and Hg, MeHg, C, N, and P concentrations (Table 2) and to characterize OM molecular composition using pyrolysis-gas chromatography-mass spectrometry. A detailed description of the different analytical methods has been published elsewhere (9).

### Determination of Hg(II) methylation rate constants in molybdate-amended sediments.

Sediment slurries were prepared by adding 30 ml of wet sediment to 30 ml of its overlying water with a range of different molybdate concentrations, from 0.1 mM to 1 mM. The concentration of molybdate was therefore set to be between 2 and 20 times higher than the prevailing sulfate concentration in the sediment overlying the water in order to approach complete inhibition of sulfate reduction. After 2 h of preequilibration, amended slurries were spiked with ^198^HgCl_2_ isotope tracer at close to ambient Hg(II) concentrations (19). One replicate was immediately frozen (*t*_0_) and another three replicates (*t*_f_) were incubated for 24 h in the glove box at 18°C and subsequently frozen. Hg(II) and MeHg were extracted from 200 mg of sediment using 7 ml of 6 N HNO_3_ with a 4-min microwave treatment at 80 W. The remaining particles were removed afterwards by centrifugation. Shortly thereafter, the extracts were buffered at pH 4, and isotopic-enriched Hg species Me^199^Hg and ^201^Hg(II) were added. The Hg(II) and MeHg species were ethylated with sodium tetraethyl borate, recovered in isooctane, and analyzed by species-specific isotope dilution using capillary gas chromatography-inductively coupled plasma mass spectrometry (GC-ICPMS) (67). Each sample was injected in triplicates, and blanks were used for contamination control. Hg(II) methylation rate constants (*k_m_*) were calculated from the initial and final concentrations of the formed MeHg species derived from the enriched isotope ^198^Hg (Me^198^Hg) after isotopic pattern deconvolution (67) and assuming a pseudo-first-order rate law. The Isotope Program in the Office of Nuclear Physics of the United States Department of Energy, Office of Science, supplied the isotopes used in this study.

### Bacterial community composition.

DNA was isolated from 0.2 g soil (wet weight) using the PowerSoil DNA isolation kit (Mo Bio Laboratories, Carlsbad, CA, USA). Bacterial primers 341F (5′-CCTACGGGNGGCWGCAG-3′) and 805R (5′-GACTACHVGGGTATCTAATCC-3′) targeting the 16S rRNA gene (68) were used for PCR amplification, where each sample was run in duplicates and 20 cycles were performed. The resulting PCR products were 100-fold diluted, and 1 μl of each of the diluted replicates was pooled and used for 10 additional cycles of amplification with barcoded primers as previously described (69). All PCRs were conducted in 20-μl volumes using 1.0 U Q5 high-fidelity DNA polymerase (New England BioLabs [NEB], UK), 0.25 μM primers, 200 μM deoxynucleoside triphosphate (dNTP) mix, and 0.4 μg bovine serum albumin (BSA). After amplicon purification with the Agencourt AMPure XP kit (Beckman Coulter, CA, USA), the final amplicon concentration was analyzed with PicoGreen as recommended by the manufacturer (Invitrogen, Carlsbad, CA, USA). Amplicons from 50 uniquely barcoded samples were pooled in equimolar concentrations, and amplicon sequencing was carried out using the Illumina MiSeq instrument with pair-end 300-bp read lengths at the SNP/SEQ SciLifeLab facility hosted by Uppsala University.

16S rRNA gene amplicon data were processed using mothur (70) according to a standard operation protocol (71). Operational taxonomic units (OTUs) were clustered at a 97% pairwise identity.

### Targeting Hg-methylating microbial communities.

The isolated DNA used for PCR amplification of the 16S rRNA gene was also used in combination with the primer pair 261F/912R. For this PCR amplification targeting the *hgcA* gene (29), we used 50 μl master mix containing 1× Phusion GC buffer, 0.2 mM dNTP mix, 5% dimethyl sulfoxide (DMSO), 0.1 μM each primer with generic adaptors, 7 μg/μl BSA, 4 μl extracted DNA template, and 1.0 U Phusion high-fidelity DNA polymerase (NEB, UK). The PCR program started with an initial 2-min denaturation at 98°C followed by 35 amplification cycles (10 s at 96°C, 30 s 56.5°C, and 45 s at 72°C), and a final 7-min extension at 72°C. A second PCR was conducted to add sample-specific DNA barcodes (Table 3). These PCRs were performed in 20-μl volumes using 1× Q5 reaction buffer, 0.2 mM dNTP mix, 0.1 μM barcoded primers, diluted first PCR products, and 1.0 U Q5 high fidelity DNA polymerase (NEB, UK) with an initial denaturation of 30 s at 98°C followed by 18 cycles (10 s at 98°C, 30 s at 66°C, and 30 s at 72°C), and a final 2-min extension at 72°C. PCR products were then purified using Agencourt AMPure XP (Beckman Coulter, CA, USA) and quantified using PicoGreen according to the instructions from the manufacturer (Invitrogen, Carlsbad, CA, USA). The amplicons were then pooled in equimolar concentrations to obtain similar numbers of sequencing reads per sample. Amplicon sequencing was carried out using the Illumina MiSeq instrument with pair-end 300-bp read lengths at the SNP/SEQ SciLifeLab facility hosted by Uppsala University.

**TABLE 3:** Barcoded adaptors^*a*^

Sample name in the database	Sample name in the main text	Index sequence	
		Forward	Reverse
VALE 01	VALE 0–1	TAGATCGC	AGGAGTCC
VALE 12	VALE 1–2	TAGATCGC	CATGCCTA
L 01	LS 0–1	TAGATCGC	AGCGTAGC
L 12	LS 1–2	TAGATCGC	CAGCCTCG
V 01	V0_1	TAGATCGC	TGCCTCTT
M 01	M 0–1	TAGATCGC	GGTATAAG
M 12	M 1–2	TAGATCGC	CAGCTAGA
LOTS 12	LOTS 1–2	TAGATCGC	TAGGCAAG
STRAN 01	STR 0–1	TAGATCGC	GTAGAGAG

a Used with the hgcA_261F (CGGCATCAAYGTCTGGTGYGC) and the reverse primer hgcA_912R (GGTGTAGGGGGTGCAGCCSGTRWARKT).

Since the length of the *hgcA* gene PCR product (680 bp) exceeded the total read length of the sequencing run (600 bp), only the forward amplicon was used for downstream data analysis. Bad-quality reads, adapters, and primer sequences were first removed (72, 73). Version 8.0 of the usearch software was used to truncate (-fastx_truncate), dereplicate (-derep_prefix), and then sort and remove singletons (-sort_by_size -minsize 2) (74). The obtained set of reads was then clustered into OTUs using cd-hit-est with an 80% similarity threshold (75). The original cleaned reads were then mapped to the representative sequences of the obtained clusters to generate a count table, using usearch (-usearch_global). The database used for the annotation of the sequences is based on the sequences published elsewhere (31).

### Phylogenetic analyses.

The *hgcA* gene sequences of this study and those previously published (31) were used to generate a hidden Markov model (HMM) with HMMER (76), and this was used to mine Deltaproteobacteria from the Integrated Microbial Genomes (IMG) database of the Joint Genome Institute (JGI). The sequences where adequately curated and the taxonomy homogenized using taxtastic (https://github.com/fhcrc/taxtastic) and R-package taxize (77). The obtained protein sequences were then aligned with MUSCLE v3.8.1551 (78). The alignment was trimmed to the size of the amplicon, and a tree was generated using RAxML v.8.2.4 (79) with the PROTGAMMLGF model and autoMR to choose the necessary bootstrap number (750). Paralogs were manually removed. The tree and the corresponding alignment were used to generate a reference package for pplacer (80), and then guppy was used to classify the sequences with a likelihood threshold of 0.8.

### Statistical analyses.

Statistical analyses were conducted using R 3.3.2 (https://www.r-project.org/). Samples were grouped with hierarchical clustering to visualize the relationships between community compositions of the samples, and the differences in community compositions observed with 16S rRNA gene and *hgcA* functional gene were compared with permutational multivariate analyses of variance (PerMANOVAs) using the function adonis and Procrustes tests of the package vegan (81).

Similarities between the bacterial community compositions and the Hg(II)-methylating community compositions of each lake at the two different depths were assessed by NMDS using the package vegan (81) and the function vegdist, with the Bray-Curtis coefficient used as a dissimilarity measure.

Redundancy analysis (RDA) was performed using the rda function in R package vegan (81) to explore the relationships between Hg(II)-methylating community composition and (i) OM composition, which was determined by Py-GC/MS, and (ii) bacterial community composition. Prior to this analysis, the relative abundances of pyrolytic compounds were log transformed in order to fit the model assumptions. The OTU tables (explanatory variables) were scaled and centered. RDA seeks a series of linear combinations of the explanatory variables that best explain the variation in the response matrix [i.e., Hg(II)-methylating bacteria] (82) but requires the number of explanatory variables to be below or equal to the number of observations (i.e., number of sediment samples, 9) and the explanatory variables to not be intercorrelated.

For the RDA of Hg(II)-methylating community composition (response matrix, 9 observations) and OM composition (explanatory matrix), the 110 identified pyrolytic organic compounds, previously characterized (9), were first grouped into 47 groups (Fig. S6) on the basis of similarity in the molecular structure within the 12 identified OM classes [i.e., carbohydrates, *N* compounds, chitin, *n*-alkenes, *n*-alkanes, alkan-2-ones, phenols, lignin, chlorophyll, steroids, hopanoids, and (poly)aromatics] (9). A subset of 9 of the 47 groups of pyrolytic organic compounds were then selected on the basis of two criteria: (i) to have a subset for the RDA which includes a group of OM compounds from the different and identified sources (terrestrial, phytoplanktonic, and microorganisms) and of different degradation statuses and (ii) to have limited intercorrelations within the subset of pyrolytic organic compound groups (intercorrelations between the 47 groups of pyrolytic organic compounds are shown in Fig. S6). RDA was performed using the subset of pyrolytic organic compounds, and intercorrelations between those variables were inspected by computing the variance inflation factor (VIF). The pyrolytic organic compounds with a VIF of >15 in the RDA model were excluded, leading to a final RDA model 1 including a subset of 5 pyrolytic organic compounds (phytene, prist-2-ene, guaiacol, indoles, and C_11–14:1_).

The correlation of NMDS structure of Hg(II)-methylating microbial communities and bacterial community composition (NMDS model 2) was tested by permutation tests (environmental fitting test, envfit function, 999 permutations) (data not shown). We selected among the most correlated explanatory bacterial OTUs to perform RDA model 2 (Syntrophobacterales, Acidobacteria_Gp15, Holophagales, Fibrobacterales, Rhizobiales, Chlorobiales, and Hydrogenophilales) (Fig. 7).

Graphics were built in R 3.2.4 (83) and modified to fit the journal requirements with Inkscape 0.92 (https://inkscape.org/es/).

### Accession number(s).

All sequence data have been deposited to the ENA Sequence Read Archive under accession number PRJEB20960.

## Supplementary Material

Supplemental file 1
