# Supplementary material for: Methanogens and Iron-Reducing Bacteria: the Overlooked Members of Mercury-Methylating Microbial Communities in Boreal Lakes
Source: Appl Environ Microbiol. 2018 Nov 15;84(23):e01774-18. doi: 10.1128/AEM.01774-18 (PMC6238055; doi:10.1128/AEM.01774-18)

Methanogens and iron-reducing bacteria: the overlooked members of mercury methylating microbial communities in boreal lakes

Running tittle: **Overlooked mercury methylators in boreal lakes**

Andrea G. Bravo[a,b+, #], Sari Peura[a,c ,+], Moritz Buck[a], Omneya Ahmed[a], Alejandro Mateos-Rivera[a], Sonia Herrero Ortega[a], Jeffra K. Schaefer[d], Sylvain Bouchet[e], Julie Tolu[f], Erik Björn[e], Stefan Bertilsson[a, #]

[a] Department of Ecology and Genetics, Limnology and Science for Life Laboratory, Uppsala University, Norbyvägen 18D, SE-75236 Uppsala, Sweden.

[b] Department of Marine Biology and Oceanography, Institut de Ciències del Mar, Consejo Superior de Investigaciones Científicas, E08003 Barcelona, Catalunya, Spain.

[c] Department of Forest Mycology and Plant Pathology, Science for Life Laboratories, Swedish University of Agricultural Sciences, Almas Allé 8, SE-756 51 Uppsala, Sweden.

[d] Department of Environmental Sciences, Rutgers University, 14 College Farm Rd, New Brunswick, 08901, New Jersey, USA.

[e] Department of Chemistry, Umeå University, Linnaeus väg 10, SE-90187 Umeå, Sweden.

[f] Department of Ecology and Environmental Science, Umeå University, SE-90187 Umeå, Sweden.

[+] These authors contributed equally to the development of this work.

[#]Andrea G. Bravo: jandriugarcia@gmail.com

[#]Stefan Bertilsson: stebe@ebc.uu.se

Supplementary Figure S1. Rarefaction curve of the *hgcA* genes. The x-axis represents the number of collected samples while the y-axis represents the total OTUs detected in this study.

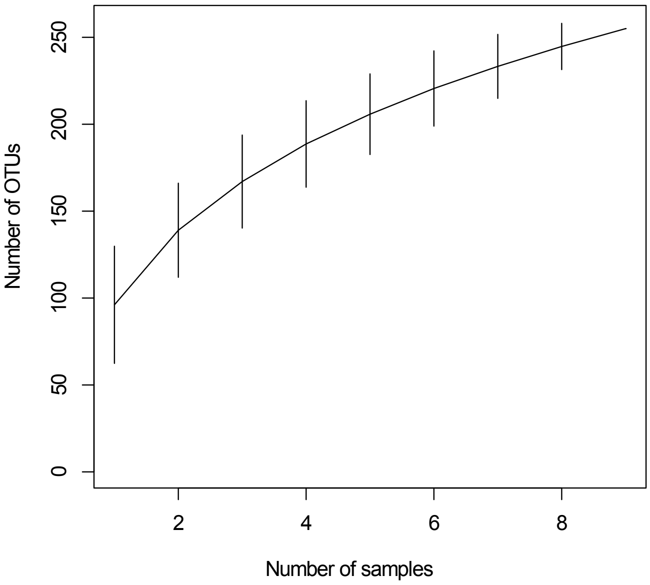

Supplementary Figure S2. Phylogenetic tree and abundance of *hgcA* functional gene OTUs. The outside colors illustrate the abundance of the sequences (total number of 78 642 sequences).

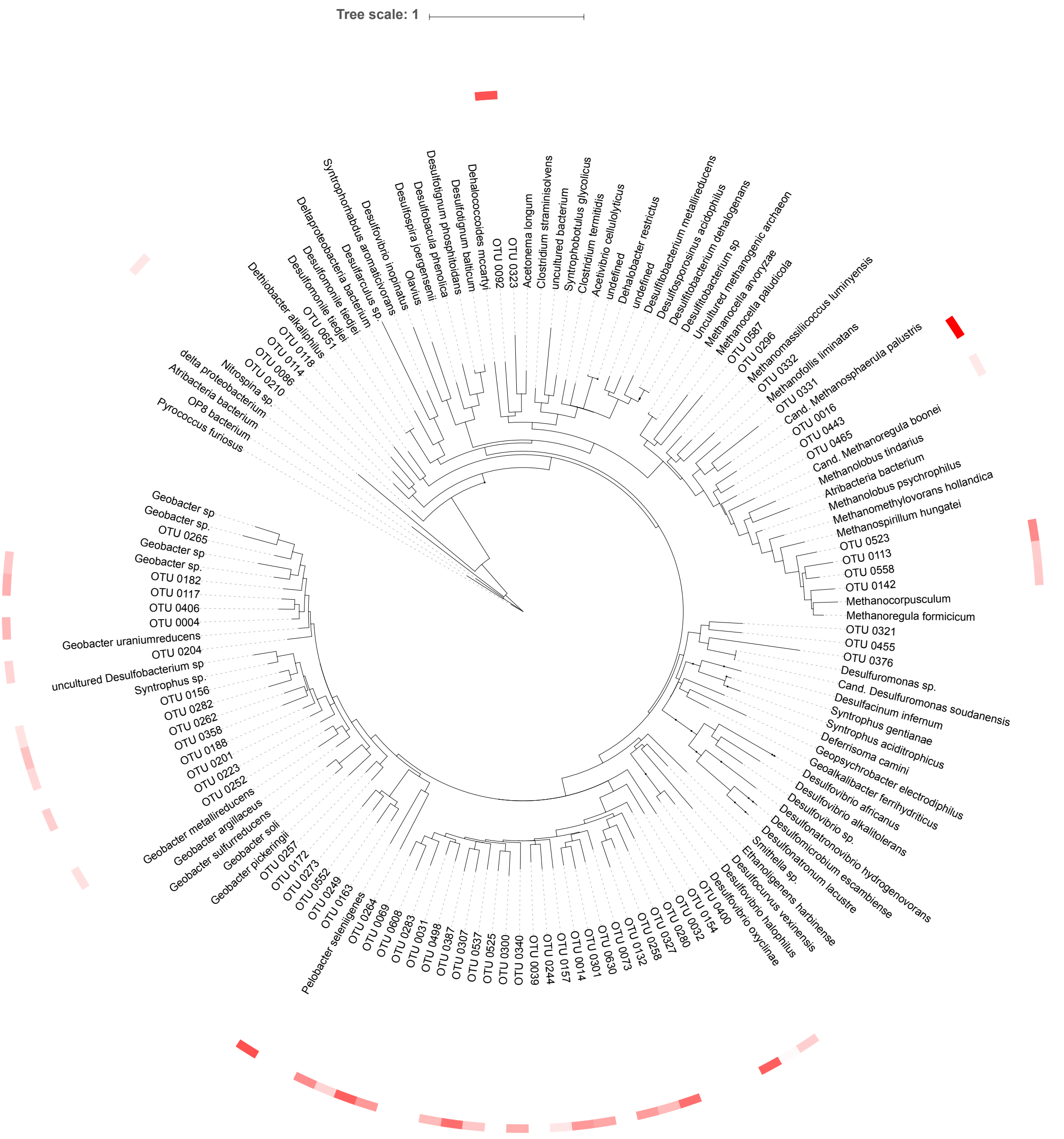

Supplementary Figure S3. NMDS (2D stress = 0.04) plot visualizing the composition of the bacterial community based on Bray-Curtis distances of the 16S rRNA gene data.

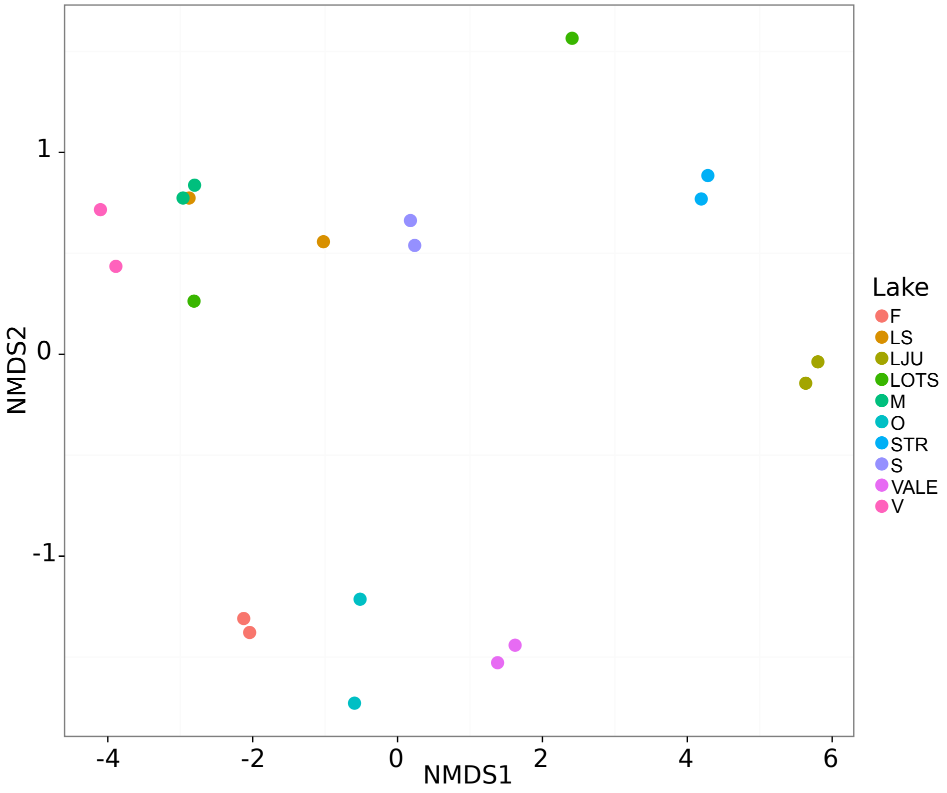

Supplementary Figure S4. Distribution of abundance at phylum-level (except *Proteobacteria* which is shown at class level) across the studied boreal lake sediments.

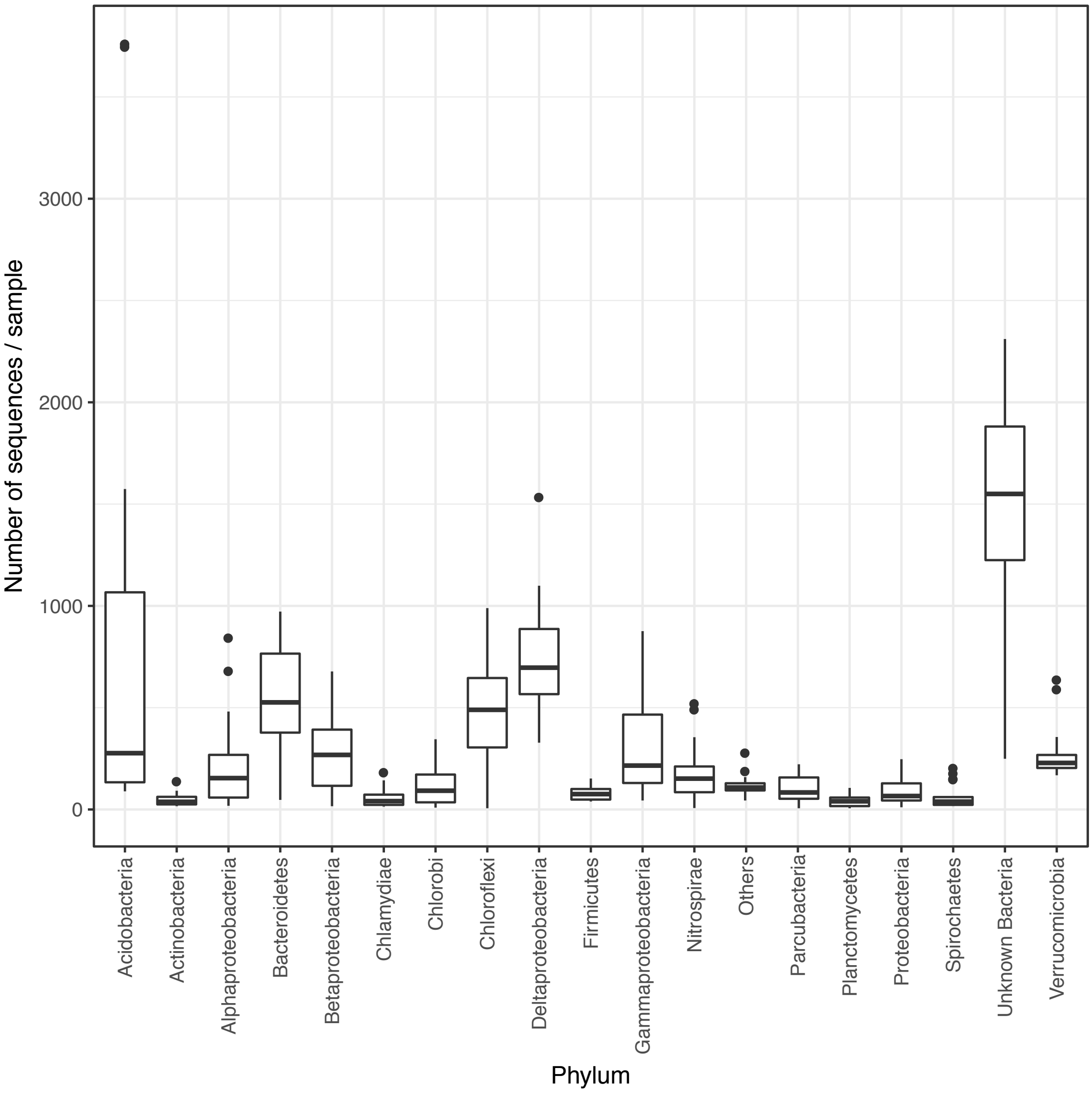

Supplementary Figure S5. NMDS plot of procrustes rotation of the Bray-Curtis distance matrices of 16S rRNA gene and *hgcA* gene data. Points represent sample ordination based on 16S rRNA gene data with arrows illustrating the position of corresponding *hgcA* gene sample.

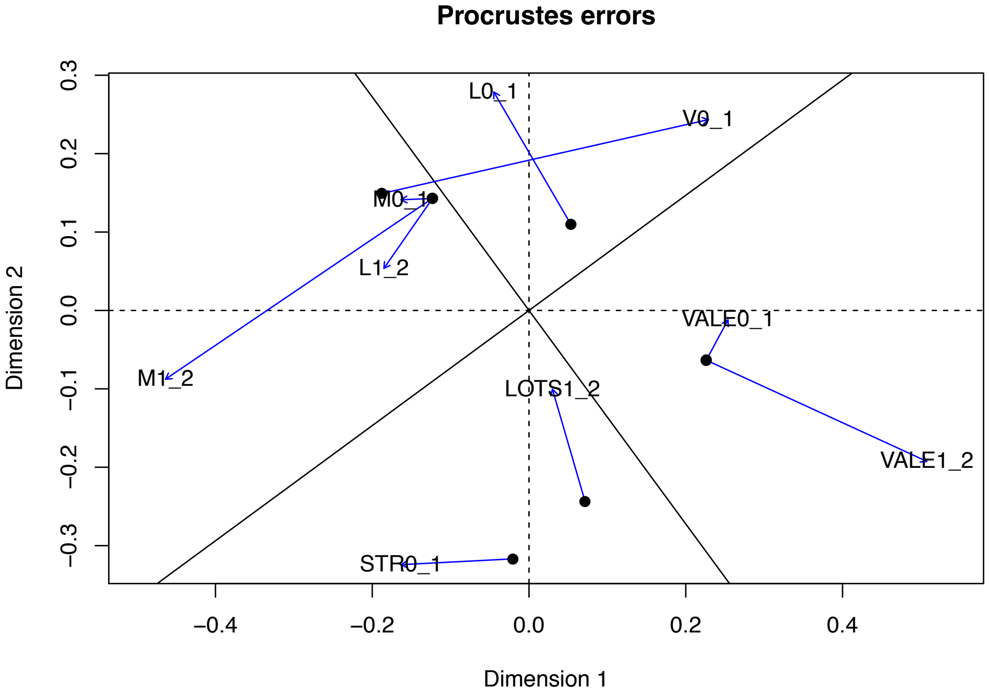

Supplementary Figure S6. Significant ($p < 0.05$) correlations between different OM compounds (colored by classes). The ellipses have their eccentricity parametrically scaled to the Pearson correlation value (narrower ellipses represents higher correlation values). The orientation of the ellipse indicates negative (red) or positive (blue) correlations (scale on the right).

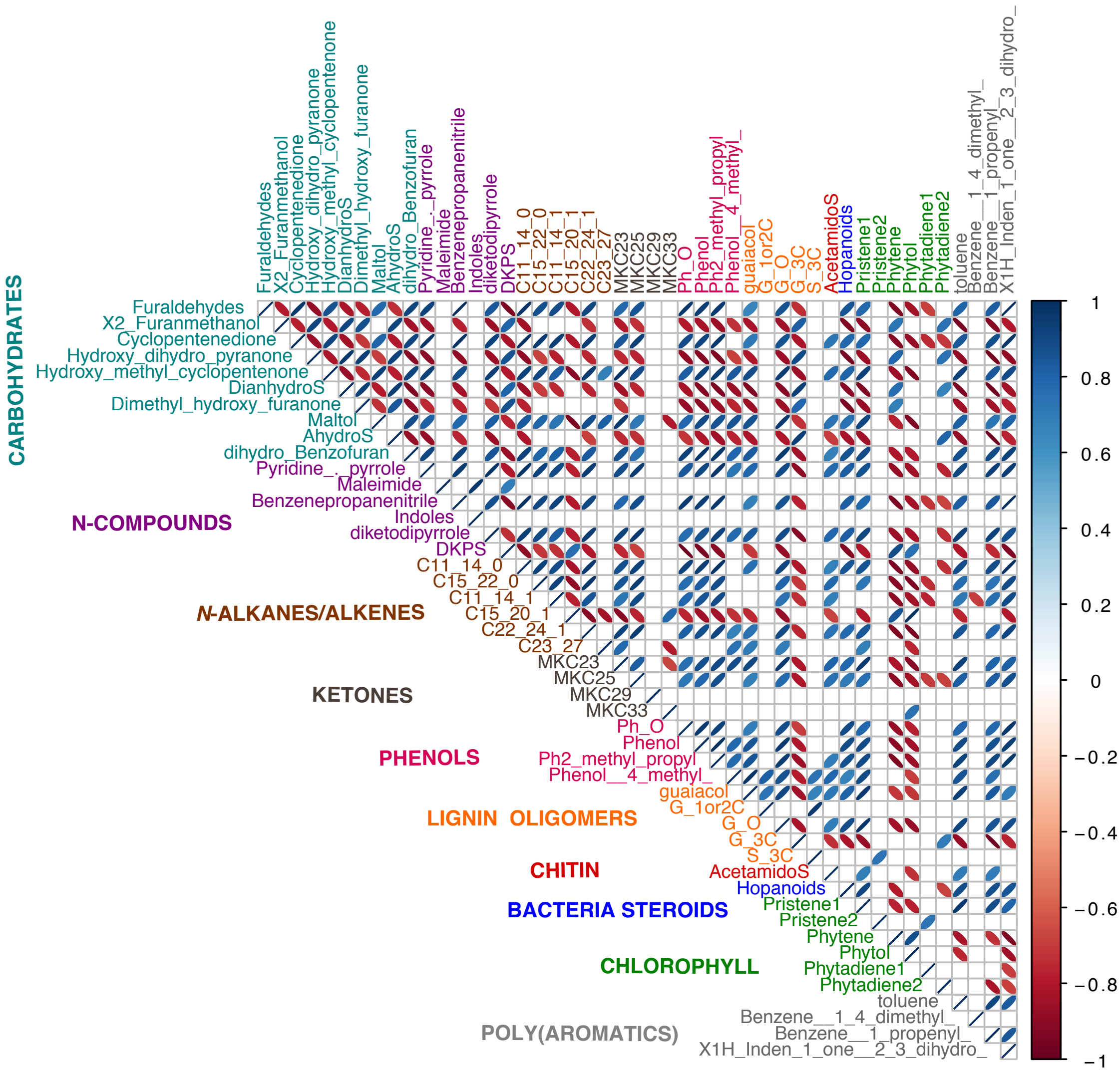

Supplement: Supplemental file 1 [file zam023188863s1.pdf]
